# Dementia Awareness Challenges in Sub-Saharan Africa: A Cross-Sectional Survey Conducted Among School Students in Ghana

**DOI:** 10.1177/15333175211055315

**Published:** 2022-01-05

**Authors:** Susanne Spittel, Elke Kraus, André Maier

**Affiliations:** 1 Charité - Universitätsmedizin Berlin, corporate member of Freie Universität Berlin and Humboldt Universität zu Berlin, Department of Neurology, Berlin, Germany; 2 Universität Bremen, Department of Health Care Research, Institute for Public Health and Nursing Research, Bremen, Germany; 3 38961 Alice-Salomon University of Applied Sciences, Berlin, Germany

**Keywords:** dementia awareness, knowledge, Sub-Saharan Africa, belief in witchcraft

## Abstract

The survey focuses on identifying dementia awareness challenges among Ghanaian school students. Data were generated in a cross-sectional survey (n = 1137). 9.3% of school students showed dementia awareness whilst the community respondents, representing both higher age and level of education, showed greater awareness (32.2%, *P* < .001). 45% of respondents believed in witchcraft and 57% were afraid of potentially being harmed by witchcraft. Age and education did not influence people’s belief in witchcraft. Moreover, dementia symptoms were often mistaken for witchcraft, especially by those who had encountered a person accused of witchcraft: “swearing at others” (24%), displaying “memory loss” and “confused speech” (22%), “forgetfulness” and who was seen “roaming around” (19%). Lack of dementia awareness was particularly evident among school students whereas belief in witchcraft was similar in both respondent groups. There was a correlation between low dementia awareness rates and misinterpretation of dementia symptoms with attribution to witchcraft.

## Introduction

The number of people living with dementia is on the increase worldwide. This development is particularly evident in low and middle-income countries (LAMIC).^1,2^ In Africa, the absolute number of people with dementia is low, but we are facing a rapid proportionate increase by more than 200%.^2^ It is estimated that by 2050, the number of people with dementia will have doubled in high income countries, and more than trebled in LAMIC.^1^ The improvement of survival rates in Sub-Saharan African countries (SSA, ie Nigeria, Democratic Republic of Congo, Senegal, Central African Republic, Tanzania, Zambia, and Kenya) will lead to an increase in life expectancy and a decline in mortality rates. Accordingly, the estimated number of older people living with and without dementia in these countries will rise.^3^ Currently, in Africa, prevalence of dementia in people aged 50+ is estimated to be about 2.4%, with variances from 1% to 10% among SSA-countries.^3,4^ Besides, there is a lack of awareness and knowledge on dementia so that persons afflicted by this and any other neurological disorder are experiencing stigmatization, discrimination and/or exclusion from society,^5-7^ as are their families, friends, and healthcare providers.^5,8^ In SSA, such stigmatization and negativity towards people with cognitive impairment is highly prevalent,^9-15^ and this creates a distance between patients and the general community, at times resulting in exclusion from society. Persons with cognitive disorders are often kept behind locked doors^10,13^ or even chained and tied up or subjected to physical violence.^14,16^ When elucidating behaviors towards persons with dementia or other cognitive impairments in SSA, beliefs in the supernatural (eg witchcraft, sorcery, and evil spirits) play an important role as these determine to a high degree people’s notions of the underlying causes of diseases in general, and of mental disorders in particular.^11,13,17,18^ Against this background, the level of awareness of age-related diseases such as dementia appears to vary among the population. For the purpose of this study, the term *dementia awareness* is defined as follows: a person who, at one point, has come across the words “dementia” or “Alzheimer’s disease” in any form is considered a person with dementia awareness. Knowledge on dementia, on the other hand, is constituted when a person declares to know what dementia or Alzheimer’s disease actually is. To our knowledge, most dementia awareness studies address the perception of dementia as a disease and the stigmata attached to it, and often pursue a qualitative approach. As for Ghana, little research has been conducted into dementia awareness.^4,10,19,20^

## Aim

In the light of the above, this survey sought to identify the challenges associated with dementia awareness in Ghana. We interviewed school students and members of the general community as to their familiarity with dementia. Our objective was to find out whether a lack of dementia awareness and a lack of understanding of the behavior displayed by people with dementia is in any way correlated to the process of branding the afflicted as “witches” or “wizards.” We hypothesize that the reason for misinterpreting the behavior of patients with dementia is a lack of knowledge on the signs and symptoms of dementia rather than an inherent belief in witchcraft alone.

## Material and Method

### Study Design

A standardized cross-sectional questionnaire-based survey was conducted in schools, churches, and villages in Ghana between 2015 and 2017. The investigation was reported according to STROBE criteria.^21^

### Participants and Setting

The recruitment of participants took place in several stages. Initially, the questionnaires were handed out in schools so as to reach the younger generation, that is, school students. Any one of the attending student was eligible for participation. To complement the data pool on dementia awareness, additional questionnaires were handed out to members of the general community, that is, at churches and in villages. Ghanaian residency was an inclusion criterion for this survey. The composition, dispatch, and collection of the questionnaire were realized in cooperation with the competent local non-governmental organization (NGO) Alzheimer´s and Related Disorders Association of Ghana (ARDAG).

### Protocol Approval and Registrations

Ethical approval for this study was obtained from the Research and Ethics Committee of the German Institute for Health Care Research (Deutsche Gesellschaft für Pflegewissenschaften e. V.). The participants were informed about the purpose of the study and assured of the confidential nature of data collection (anonymization of personal information). The data were coded whilst their geographical origin was disclosed.

### Variables and Data Source

The original questionnaire was designed in 2012 and 2013 and scrutinized for suitability in a pilot study with nursing students of the University of Cape Coast (Central Region, Ghana).^22,23^ Subsequently, updates were implemented so as to consider current knowledge and understanding of dementia, witchcraft and older people’s behavior. The resulting 20-item semi-structured questionnaire was co-validated by select national experts of ARDAG. Four of the questions were based on the Alzheimer’s Disease Knowledge Scale, more particularly on items dealing with the symptomology of dementia.^24^ The final version was reviewed and passed by the Ethics Committee of the German Institute for Health Care Research. Please refer to Supplementary File 1 for textual and categorical details.

#### Socio-demographic data

Socio-demographic data included age, gender, nationality, place of birth, marital status, religion, and educational status (7 questions, Supplementary File 1).

#### Data on aging and familiarity with dementia

Three questions dealt with aging and familiarity with dementia and enquired about the respondents’ awareness and knowledge on the disease, the age of their grandparents and if these showed or had shown any symptoms of memory loss and inability to perform activities of daily life. An additional questionnaire was included to investigate about the prevalence of the aforesaid symptoms among any other family members at any point in their lives (to specify the respective relative’s age at symptom onset was optional, Supplementary File 1).

#### Data on belief in witchcraft

The section on witchcraft (6 questions, Supplementary File 1) elucidated whether the study participants and their family members held beliefs of witchcraft and whether they had actually had an encounter with a person accused of witchcraft.

Furthermore, it investigated the behaviors and situations attributed to witchcraft (eg roaming around, forgetfulness, confusion in thought and speech, memory problems, and swearing at others). The wording “a person accused of witchcraft” introduced in this context is defined as follows: a person caught in the act of “positive or negative sorcery,” a well-meaning “wizard” or healer who has lost the trust of his clients, a person with a reputation for being a “witch,” or even a person who has merely incurred the enmity of his neighbors.^25^ In Ghana, “*women who do not fall under the direct “control” of a man, or are economically successful, or childless, or without appropriate familial protection, or in competition for scarce resources, can all too easily become targets for gossip and jealousy*” and most frequently targeted by accusations of witchcraft.^26^ The final questions enquired about the level of attention that needed to be given to the elderly, age-related diseases, and witchcraft. There was also a free text field for personal comments (Supplementary File 1).

### Data Analysis

The data obtained were analyzed using SPSS version 24. Descriptive results were calculated as frequencies (%), means, medians, standard deviation (±), minimum (Min), and maximum (Max). Metric results were expressed as means when distribution was normal and as medians when distribution was non-Gaussian. Socio-demographic variables, for example, educational status and religion as well as the age of the participants’ family members with dementia symptoms were grouped. Group differences between socio-demographic characteristics and dementia awareness or the belief in witchcraft were tested by Chi-Square Test and Fisher’s Exact Test. Differences in the non-grouped age on a metric scale were tested using the Mann–Whitney U test. Significance differences were calculated at *P* < .05. Answers to the 3 open questions were categorized and coded by content, including demographical aspects, awareness and understanding of dementia, and the issue of stigmatization and discrimination.

## Results

A total of 1137 participants completed the questionnaire. The majority were school students (main cohort, n = 979, 86%) and a smaller proportion were members of the general community (comparative cohort, n = 158, 14%).

### Socio-Demographic Characteristics

60.7% of participants (n = 686) were female, 39.3% (n = 445) were male. Most participants were still in school (91.6%) and Christians (92.5%). The overall median age (in years) was 17.0 (±8.5, mean: 19.6), with a range from 10 to 80. At that, the school students were significantly younger than the members of the community (17.0 years vs 30.5 years, *P* < .001). The socio-demographic characteristics are summarized in Table 1.

**Table 1.** Socio-Demographic Characteristics of Participants.

Variable	Type	Total, n = 1137	Students, n = 979	Community, n = 158	*P*-value
Gender	Female, n (%)	686 (60.7)	609 (62.3)	77 (50.0)	.003
	Male, n (%)	445 (39.3)	368 (37.7)	77 (50.0)	
Age	Years, median (SD, range)	17.0 (8.5, 10-80)	17.0 (1.6, 13-29)	30.5 (.8, 10-80)	<.001
Religion	Christianity, n (%)	953 (92.5)	806 (91.8)	147 (96.7)	<.001
	Islam, n (%)	69 (6.7)	65 (7.4)	4 (2.6)	
	Traditional, n (%)	2 (.2)	2 (.2)	0 (0)	
	Other religion, n (%)	6 (.6)	5 (.6)	1 (.7)	
Educational status	No education, n (%)	5 (.4)	0 (0)	5 (3.4)	<.001
	Primary school education, n (%)	1032 (91.6)	979 (100)	53 (35.8)	
	Highschool education, n (%)	52 (4.6)	0 (0)	52 (35.1)	
	University graduate, n (%)	38 (3.4)	0 (0)	38 (25.7)	
Have heard of dementia	Yes	135 (12.4)	87 (9.3)	48 (32.2)	<.001
Knowledge on dementia	Yes	123 (11.3)	75 (8.0)	49 (32.5)	<.001
Family members with dementia symptoms	Yes	177 (16.6)	129 (14.0)	48 (32.3)	<.001
Believe in the existence of witchcraft	Yes	508 (44.9)	430 (44.1)	79 (49.4)	.228
Believe in the power of witchcraft	Yes	638 (57.1)	542 (56.5)	96 (60.8)	.341
Contact with a person accused of witchcraft	Yes	475 (42.5)	428 (44.5)	47 (30.1)	.001
Family members believe in witchcraft	Yes	569 (51.1)	484 (50.4)	85 (55.6)	.258
See witchcraft as important part of culture	Yes	123 (11.1)	82 (8.6)	41 (27.3)	<.001
More attention needs to be given to older people	Yes	1003 (91.5)	855 (90.9)	148 (95.5)	.033
More attention needs to be given to age-related diseases	Yes	870 (84.5)	729 (82.8)	141 (94.0)	<.001

Abbreviations: n number of participants, SD standard deviation.

Notes: Other religions: Judaism, Hinduism, and others; Difference of frequencies between the two groups were assessed by chi-square test and between metric-data by Mann–Whitney U test; a *P*-value <.05 was considered to be significant. Significant differences were measured between the youngsters and the general community; Students: school students; Dementia awareness is defined by whether respondents have ever heard of or read about the term dementia or Alzheimer’s disease; Knowledge on dementia means whether respondents reported knowing or not knowing what dementia or Alzheimer’s is.

### Aging and Familiarity With Dementia

The participants were asked about any relatives who had either lived to old age or were still alive and of advanced age. The mean age of the respondents’ grandparents was 80.6 years (±16.1). One finding was that grandmothers were significantly younger—almost 3 years—than grandfathers: their respective life expectancy was 79.4 years and 82.0 years (*P* < .001). In total, 7.8% of grandparents (n = 257), whether alive or deceased, showed or had shown symptoms of memory loss or inability to perform activities of daily life. Symptomatic grandparents were significantly older than grandparents with no such symptoms (83.1 years vs 80.7 years, *P* < .05). Females were more likely to show symptoms than males (9.1% (n = 155) and 6.5% (n = 102), respectively, (*P* = .005)). In a further step, the respondents identified any family members at all who were or had been affected by memory loss and inability to perform activities of daily life (16.6%; n = 177). The mean age of these family members was 60.3 years (±26.8), with a range from 14 to 150. Most of them were less than 60 years old (50.9%, n = 83). Only 12.4% (n = 87) of respondents were aware of dementia or dementia-related terms (as per our definition); and only 11.3% (n = 123) had any knowledge on the disease (Table 1).

**Figure 1.**
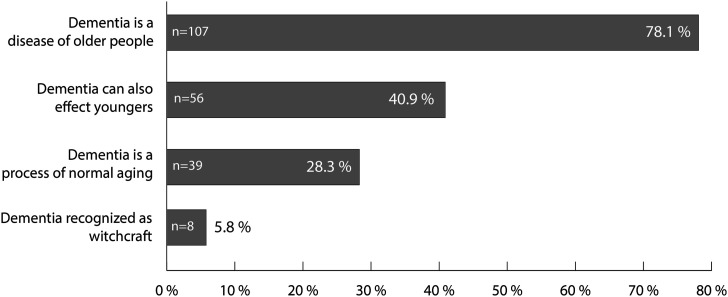
Perceptions of dementia of those participants who stated to have knowledge on the disease. n = number of participants.

Participants with a higher level of education displayed greater awareness and knowledge on dementia (*P* < .001; Phi/Cramer’s V = .259). Moreover, there was a significant correlation between the participant’s age and their awareness and knowledge on dementia. Participants with some knowledge on dementia were also older (mean age of 24.4) than those who had no knowledge on the disease at all (mean age of 18.7; *P* < .001). Dementia awareness was significantly higher (32.2%) among the general community than among school students (9.3%, *P* < .001, Table 1). Respondents whose family members showed symptoms of “memory loss” or “inability to perform activities of daily life” were more aware of dementia than respondents whose family members were not afflicted by the disease (27.1% vs 9.5%, *P* < .001, Table 1). At 38.5% (*P* < .001), the share of respondents that showed dementia awareness was higher in families where the afflicted were 60 years or older. Furthermore, participants with knowledge on dementia regarded dementia as a “disease of older age” (78.1%). 40.9% of these knew that dementia can also affect younger people, 28.3% saw dementia as a normal part of aging, and 5.8% related dementia to witchcraft (Figure 1).

Awareness of dementia was significantly higher among those respondents who regarded witchcraft as an important part of their culture (38.1% vs 9.2%, *P* < .001), and also among those who felt that in Ghana, both older people and the subject of witchcraft require a greater degree of attention (Table 2).

**Table 2.** Characteristics of Participants Who are Familiar With Dementia versus Those Who are Not.

Variable	Type	Have heard of dementia, n = 1085			Knowledge on dementia, n = 1093		
		Yes	No	*P*-value	Yes	No	*P*-value
Gender	Female, n (%)	77 (11.8)	573 (88.2)	.510	71 (10.9)	582 (89.1)	.625
	Male, n (%)	57 (13.3)	373 (86.7)		52 (12.0)	382 (88.0)	
Age	Years, mean (SD)	24.4 (13.0)	18.7 (7.1)	<.001	25.5 (13.6)	18.7 (7.1)	<.001
Educational status	No education, n (%)						
	Primary education, n (%)	96 (10.6)	811 (98.4)	<.001	82 (9.0)	832 (91.0)	<.001
	Highschool education, n (%)	11 (22.0)	39 (78.0)		11 (21.6)	40 (78.4)	
	University graduate, n (%)	25 (69.4)	11 (30.6)		27 (73.0)	10 (27.0)	
Family member with dementia symptoms	Yes, n (%)	46 (27.1)	124 (72.9)	<.001	42 (25.0)	126 (75.0)	<.001
	No, n (%)	81 (9.5)	774 (90.5)		75 (8.7)	789 (91.3)	
Believe in the existence of witchcraft	Yes, n (%)	58 (12.1)	423 (87.9)	.853	63 (12.9)	424 (87.1)	.152
	No, n (%)	75 (12.5)	524 (87.5)		61 (10.1)	540 (89.9)	
Believe in the power of witchcraft	Yes, n (%)	72 (11.8)	540 (88.2)	.401	73 (11.9)	538 (88.1)	.629
	No, n (%)	62 (13.7)	392 (86.3)		50 (10.8)	412 (89.2)	
Contact with a person accused of witchcraft	Yes, n (%)	57 (12.5)	399 (87.5)	.851	51 (11.1)	407 (88.9)	1.000
	No, n (%)	74 (12.1)	540 (87.9)		69 (11.1)	550 (88.9)	
Family members believe in witchcraft	Yes, n (%)	80 (14.8)	459 (85.2)	.004	79 (14.5)	467 (85.5)	.001
	No, n (%)	49 (9.4)	475 (90.6)		42 (8.0)	484 (92.0)	
Witchcraft seen as important part of culture	Yes, n (%)	43 (38.1)	70 (61.9)	<.001	41 (35.3)	75 (64.7)	<.001
	No, n (%)	87 (9.2)	859 (90.8)		78 (8.2)	872 (91.8)	
More attention needs to be given to older people	Yes, n (%)	124 (13.0)	833 (87.0	.052	116 (12.0)	850 (88.0)	.040
	No, n (%)	6 (6.7)	84 (93.3)		5 (5.6)	85 (94.4)	
More attention needs to be given to age-related diseases	Yes, n (%)	109 (13.1)	725 (86.9)	.066	103 (12.2)	740 (87.8)	.067
	No, n (%)	13 (8.4)	141 (91.6)		12 (7.7)	143 (92.3)	
More attention needs to be given to witchcraft	Yes, n (%)	48 (16.9)	236 (83.1)	.003	45 (16.1)	235 (83.9)	.002
	No, n (%)	75 (10.3)	653 (89.7)		69 (9.3)	673 (90.7)	

Abbreviations: n, number of participants; SD, standard deviation.

Notes: Dementia awareness is defined by whether respondents have ever heard of or read about the term dementia or Alzheimer’s disease; Knowledge on dementia means whether respondents reported knowing or not knowing what dementia or Alzheimer’s is; *P*-values obtained using t-test for comparison between means, and chi-square test for comparison between frequency data; a *P*-value <.05 was considered significant; family members with dementia symptoms means: family members showing symptoms of memory loss and forgetfulness.

### Belief in Witchcraft

**Figure 2.**
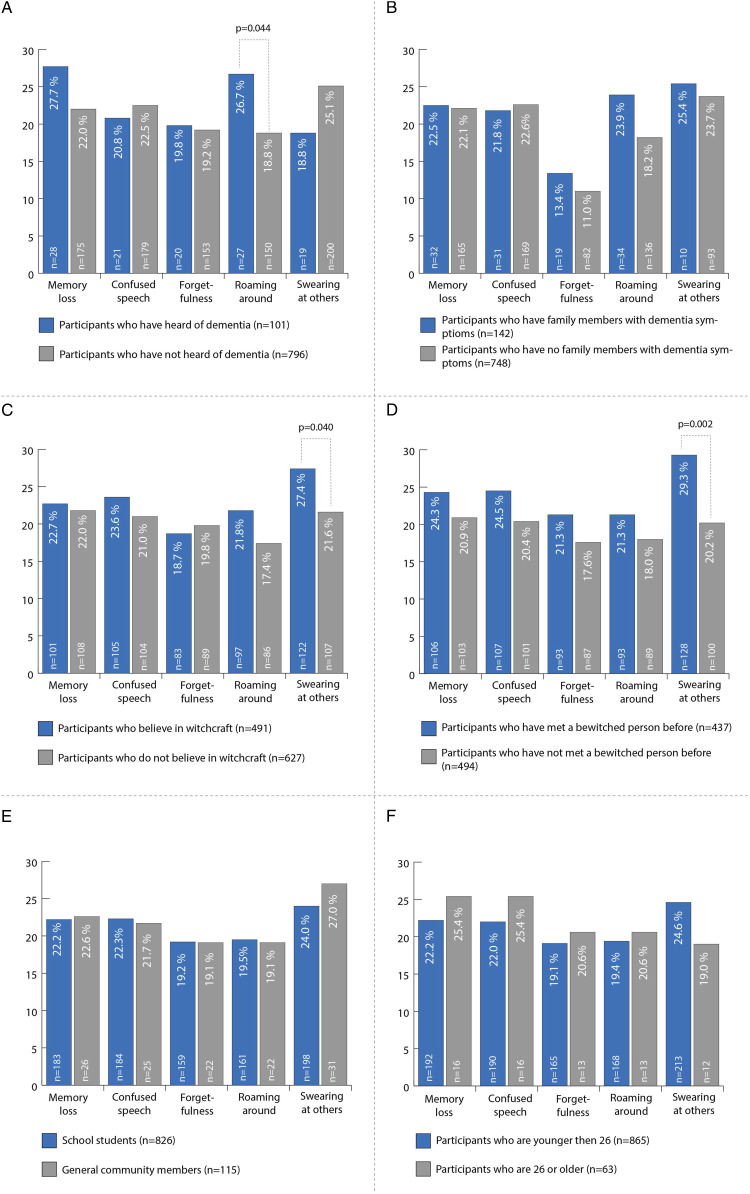
Behavior associated with witchcraft. Significant differences were assessed by Chi-Square test; a *P*-value <.05 was considered significant. n = number of patients.

The survey results showed that 44.9% of the participants (n = 508) held a belief in witchcraft. A larger share, that is, 57.1% (n = 638), said that witchcraft worked and had the potential to cause harm. There were no significant differences in the rates for belief in witchcraft between the respondent school students and community members (44.1 vs 49.4%, *P* = .228), nor between respondents from urban and rural areas (42.6% vs 46.6%, *P* = .423), nor between female and male participants (43.9% vs 46.5%; *P* = .392). Age did not matter with regard to the participants’ belief in witchcraft (20.1 years [mean age of believers] vs 19.1 years [mean age of non-believers]). Furthermore, 51.1% (n = 569) revealed that some family members believed in witchcraft, and 47.9% (n = 545) had already been in contact with a person allegedly exercising witchcraft. Participants who had met such a person were more likely to believe in witchcraft than participants who had not done so (59.3% vs 40.7%; *P* < .001). Likewise, participants with exposure were more likely to be afraid of potentially being harmed by witchcraft (70.8% vs 29.2%; *P* < .001). School students reported to have had significantly more encounters with a person accused of witchcraft than respondents from the general community (44.5% vs 30.1%, *P* < .001). Moreover, participants attributed signs and mannerisms that are also manifest in patients with dementia (“swearing at others” (24.3%, n = 229), “confused speech” (22.2%, n = 209), “memory loss” (22.2%, n = 209), “seen roaming around” (19.4%, n = 183), or “forgetfulness” (19.2%, n = 181)) to the behavior of persons accused of witchcraft. Participants who stated to have heard of dementia were more likely to relate “memory problems” (27.7% vs 22.0%) or “roaming around” (26.7% vs 18.8%; *P* < .05) to witchcraft (Figure 2A). The likelihood of assigning symptoms and behaviors like “roaming around” (23.9% vs 18.2%), “forgetfulness” (13.4% vs 11.0%), or “swearing at others” to witchcraft was higher among participants with family members with dementia symptoms (Figure 2B) than among those with no such relatives. Moreover, participants who believed in witchcraft were more likely to attribute “swearing at others” (27.4% vs 21.6; *P* < .05), “confused speech” (23.6% vs 21.0), and “roaming around” (21.8% vs 17.4%) to witchcraft, compared with those who did not believe in witchcraft (Figure 2C). Participants with actual encounters with persons accused of witchcraft more frequently judged symptoms typically manifesting with dementia (“memory problems” (24.3% vs 20.9%), “confused speech” (24.5% vs 20.4%), “forgetfulness” (21.3% vs 17.6%), “roaming around” (21.3% vs 18.0%), and “swearing at others” (29.3% vs 20.2%; *P* < .05)) to be the behavior of persons accused of witchcraft than participants who had not had such an encounter (Figure 2D). 11.1% (n = 123) of participants saw witchcraft as an important part of their culture. This group was more likely to associate signs and symptoms of dementia such as “memory problems” (25.0% vs 22.0%), “confused speech” (25.0% vs 22.0%), “forgetfulness” (23.1% vs 19.0%), and “roaming around” (21.2% vs 19.1%) with witchcraft than the group that did not consider witchcraft an important part of their culture. School students and members of the general community almost equally attributed symptoms of dementia to witchcraft (Figure 2F). By comparison, participants of higher age (26+ years) more often regarded “memory problems” (25.4% vs 22.2%) and “confused speech” (25.4% vs 22.0%) as signs typical of persons associated with witchcraft than younger people did (<26 years, Figure 2F).

All in all, the vast majority of the respondents said that more attention should be given to older people (91.5%, n = 1003) and age-related diseases (84.5%, n = 870) in their respective countries. Ghanaian respondents who saw a need to give “older people,” “age-related diseases,” and “witchcraft” more attention reasoned that there is a lack of understanding of how older people behave in their country (eg misinterpretation of dementia as witchcraft: *“Because due to illiteracy some people in Ghana think old people are witches/wizards.”, “People should be educated that witchcraft is not responsible for the illness.”,* Supplementary File 2).

## Discussion

### Living to Older Age in SSA

According to the participants, people in Ghana do in fact live to older age. This is supported by the fact that the average age of their grandparents was 81 years. This is a remarkable finding as the 2018 World Bank Data statistics quote a life expectancy of 63.8 years for Ghana. However, these data should be treated with caution,^27^ as in Ghana, the first official registration system for births and deaths was only introduced in the 1960s (Ghana: Act No. 301, 1965). In our survey, grandmothers were found to be significantly younger than grandfathers, that is, almost 3 years (female grandparents: 79 years vs male grandparents: 82 years), a finding that does not correspond to the World Bank Data statistics either. The relevant passage states that life expectancy is 65 years for women and 62 years for men. Although this does not give ground for further assumptions, it proves the prevalence of older people in Ghanaian societies. Improved living standards and enhanced longevity may contribute to a continuing increase in the number of older people in future years.^28,29^ Ghana is among the African countries with the most rapid increase in the percentage share of older people.^30^ We furthermore found that participants identified signs and symptoms of “memory loss” or “inability to perform activities of daily life” in either their grandparents only (about 8% with a mean age of 81 years) or any other family members at all, that is, including grandparents (about 17% with a mean age of 60 years). These results could be regarded as indicative for the occurrence of dementia diseases in Ghana. Dementia prevalence of 10% among the participants’ grandparents support findings from dementia prevalence studies conducted in Africa.^3,4^ A study revealed scarcity of geriatrics experts and the relative absence of diagnostic screening for dementia in Ghana.^22,23^ We therefore conclude that dementia is not widely diagnosed and the actual prevalence and incidence of dementia may deviate even more from our findings.

The high number of younger family members with “memory loss” and “inability to perform activities of daily life” (mean age of 60 years) could be related to the high prevalence of infectious diseases in SSA which impacts the prevalence of common mental disorders (eg HIV-related dementia),^31-34^ a scenario that would be of interest for future investigations.

### Familiarity With Dementia

The results of this study reveal that there is hardly any awareness of dementia diseases in the population under investigation. The vast majority (88%) had neither heard of dementia nor displayed any knowledge thereon. Similar findings arose from a South-African and a Nigerian community survey. At 10%, the number of respondents that stated they knew what dementia actually was, was equally low.^7,35^ In these surveys, common names for dementia used by the Nigerian community were “memory loss disease,” “aging disease,” “disease of insanity,” or “forgetfulness.” Even though in this study 67% of participants had already heard about the disease, the majority of them were aged 60+.^35^ Accordingly, the respondents in our study who had heard about dementia were significantly older and had a higher level of education. Another study, this time conducted in Tanzania, revealed that neither the majority of people personally afflicted with dementia nor their caregivers actually knew of dementia and its underlying causes, and they perceived symptoms like memory loss as part of normal aging or attributed them to witchcraft.^13^ A survey of the general population conducted in the Republic of Congo revealed that dementia was not perceived as a disease among community members.^11^ We found significantly greater awareness in the general community (32%) than in school students (9%)—accordingly, in Ghana, awareness is still low. In South Africa, the various types of dementia tend not to be diagnosed in patients.^14^ Moreover, previous findings from Ghana indicated that there were hardly any mental health care specialists and dementia diseases are neither diagnosed nor treated.^22^ In our study, most participants with knowledge on dementia identified dementia as a “disease of older age” (78%). Only 41% of participants knew that dementia can also affect younger people, about one third saw dementia as part of normal aging, and about 6% related dementia to witchcraft. Generally, biomedical concepts are rarely considered to be the cause of dementia in SSA. This is a misconception that was also evident in our study findings. Participants explained that more attention should be given to older people and age-related diseases as there is a tendency to declare older people “witches” and “wizards.” Dementia is also misinterpreted as witchcraft. And all of this is due to the fact that the population is lacking in knowledge on the disease.

### Belief in Witchcraft

The concept that dementia is seen as a work of “witches” is a common problem in SSA.^5,6^ In most African cultures, there is no “name” for dementia, which ultimately results in older people being accused of exercising witchcraft to harm other people.^14,36^ Alarmingly, we also found that signs and symptoms typically manifesting with dementia were frequently seen as behaviors of “witches” and “wizards.” “Swearing at others” (24%) was most frequently linked to witchcraft. Even the more typical symptoms of dementia like “confused speech” and “memory problems” (22%), “forgetfulness” and seen “roaming around” (19%) were commonly linked to witchcraft. As confirmed by other study results, fearfulness or violence is seen to be caused by witchcraft or based on the belief that fearful/violent people are bewitched.^14^ It can be assumed that the vast majority is aware of the superstition of witchcraft as this belief is strongly held in most SSA-countries.^20^ Interestingly, in this study the participants who had already come across a person allegedly exercising witchcraft were more likely to link behaviors of people with dementia to witchcraft than those who had not had such an encounter (Figure 2). This is an alarming result reinforcing the idea that typical symptoms of dementia are also attributed to witchcraft. Especially school students who were found to be less aware of dementia (only 9% had ever heard of the disease) stated to have met a person accused of witchcraft (45% vs 30% of people in the community). Based on this, we can assume that youngsters are more likely to misinterpret people’s behavior for witchcraft as they are less able to understand the signs and symptoms of dementia. This finding is worth discussing, as the percentage share of believers in witchcraft was similar in the respondent groups of school students and community members. In view of these facts, the study reinforces the assumption that a lack of dementia awareness and an associated lack of understanding of the behavior displayed by persons with dementia result in “mistaking” them for “witches” and “wizards.”

Besides, participants, who had met a person accused of witchcraft, were significantly more likely to believe in witchcraft and to be afraid of being on the receiving end of spells and curses. Another assumption worth discussing is that less awareness of behaviors drives fear and, consequently, misinterpretation. People’s fear of persons accused of witchcraft is demonstrated by a story *of a* 72-years old Ghanaian woman, who was burned to death for being a witch. After she had died her son explained: “*Our mother was never a witch and had never suffered any mental disorder throughout her life, apart from exhibiting signs of forgetfulness and other symptoms of old age.”*^37^ Those supernatural approaches to disease primarily exist in societies dominated by great traditions and can gravely influence the perception of older people’s’ behaviors, as well as of dementia diseases as long as an understanding of such conditions is still lacking.^5,12,19,38^ In Ghana, the belief in the power of witchcraft is also widespread (57%) and, interestingly, not related to people’s age, gender or origin (urban vs rural). Notwithstanding, most respondents did not see witchcraft as an important part of their culture (only 11% do so) or believed that it should be given more attention in the future (only 28%). In this context, it was notable that respondents who had an awareness of dementia attached significantly more cultural importance to witchcraft (33%, n = 43) and were also significantly more likely to say that the subject of witchcraft should be paid more attention (39%, n = 48). It is worth discussing that people with an awareness of dementia saw the danger that a person may be wrongly accused of witchcraft, or had even come across persons with dementia that were regarded as “witches” just because they behaved differently. These respondents were also more inclined to state that more attention should also be paid to the issue of witchcraft. Similarly, the correlation between higher disease awareness and the established importance of witchcraft suggests that dementia awareness and knowledge play an important role in the perception of the disease as such. Disease awareness is considered to play an important role, as understanding of symptoms and behaviors can actually prevent superstitious perceptions of diseases and stigmatization.^5^ We may assume that as long as people believe in witchcraft this misbelief will always serve as a popular source for alternative explanations for behavioral patterns displayed by the elderly and/or patients with dementia.

### Limitations and Future Study

This research was limited to Ghana. Even though there are indicators that a lack of awareness of dementia and a belief in witchcraft both nurture misinterpretation of diseases, further research is needed to confirm results. Such investigations should be extended across Sub-Saharan Africa. Additionally, the research was limited to a few districts of Ghana. Due to various cultural differences within Ghana and variations between life in rural and urban areas, there could well be varying views taken by the population in other parts of the country under scrutiny. Answers to the topics and themes addressed in the study were collected from young people who were still in school. Although the mean age of participants (20 years) corresponds to the median age of the population in Ghana (21 years in 2015),^39^ we must assume results for the older population to be different, as was the case with the additional respondent cohort in the general community. It is not possible to generalize the results for the Ghanaian population as a whole. Especially people with a university degree could well have different opinions, as was indicated by our study results revealing significant differences in awareness and knowledge on dementia between the group with a higher level of education and school students.

## Conclusion

Even though life expectancy in Ghana is still low, the average age of the population of Ghana will increase more and more rapidly in the future, and dementia prevalence will increase accordingly. However, there is hardly any awareness of dementia diseases among school children and members of the general community in Ghana. As long as superstitious beliefs such as witchcraft flourish and a poor understanding of diseases prevails, people afflicted with dementia are carrying an extra burden, and their lives might even be in danger. In this regard, this study highlights the urgency of promoting awareness and knowledge on dementia diseases, particularly among the younger generation, but also among the broader public so that people with dementia may live a fuller life, be encouraged to participate in societal activities, and stand a chance of receiving enhanced treatment for their condition. With this goal in mind, the rate of relating mentally ill people’s behavior to witchcraft will presumably decrease over time. In a first step, people will need to be educated about the signs and symptoms of dementia diseases and related behavior so that they can come to an understanding of the characteristics of the disease. They will more likely to refrain from wrongly attributing clinical symptoms to witchcraft. Secondly, enhanced awareness of all forms of dementia (eg juvenile form of dementia) is needed to eliminate the stigma attached to it. Involving the younger generation in awareness campaigns could be one focus as this may make them more open-minded which in turn would be a starting point to initiate change. We can expect them to bring their “new” knowledge to the communities they live in and enter into a dialogue with older community members who might not have any dementia awareness at all. As young people will be the future population and leaders of their countries, policymakers and health care practitioners need to be culturally sensitive and take these health care situations into account when aiming to improve the lives of this particularly disadvantaged group of the population. Raising dementia awareness in SSA is mandatory so that people can live in a society where a person with dementia can live with dignity and is met with respect.

## Supplemental Material

sj-pdf-1-aja-10.1177_15333175211055315 – Supplemental Material for Dementia Awareness Challenges in Sub-Saharan Africa: A Cross-Sectional Survey Conducted Among School Students in Ghana Click here for additional data file. Supplemental Material, sj-pdf-1-aja-10.1177_15333175211055315 for Dementia Awareness Challenges in Sub-Saharan Africa: A Cross-Sectional Survey Conducted Among School Students in Ghana by Susanne Spittel, Elke Kraus and André Maier in American Journal of Alzheimer's Disease & Other Dementias

sj-pdf-2-aja-10.1177_15333175211055315 – Supplemental Material for Dementia Awareness Challenges in Sub-Saharan Africa: A Cross-Sectional Survey Conducted Among School Students in Ghana Click here for additional data file. Supplemental Material, sj-pdf-2-aja-10.1177_15333175211055315 for Dementia Awareness Challenges in Sub-Saharan Africa: A Cross-Sectional Survey Conducted Among School Students in Ghana by Susanne Spittel, Elke Kraus and André Maier in American Journal of Alzheimer's Disease & Other Dementias
