# Supplementary material for: Dementia Awareness Challenges in Sub-Saharan Africa: A Cross-Sectional Survey Conducted Among School Students in Ghana
Source: Am J Alzheimers Dis Other Demen. 2022 Jan 5;36:15333175211055315. doi: 10.1177/15333175211055315 (PMC10581119; doi:10.1177/15333175211055315)
Supplement: sj-pdf-1-aja-10.1177_15333175211055315 – Supplemental Material for Dementia Awareness Challenges in Sub-Saharan Africa: A Cross-Sectional Survey Conducted Among School Students in Ghana [file sj-pdf-1-aja-10.1177_15333175211055315.pdf]

Supplementary File 1. Variables and questions included in the questionnaire.

**Socio-demographic data**		
Age	Free text field	Age in years
Gender	Checkboxes	Male l female
Nationality	Free text field	
Place of birth	Free text field	
Marital status	Checkboxes	Single l Married l Divorced l Widowed l Partner
Religion	Checkboxes	Christianity l Islam l Hinduism / Buddhism l Judaism l Traditional / folk religions l Atheist / non-believer
Education	Checkboxes	No education l Primary school education l Highschool education l University graduate
**Data on ageing and familiarity with dementia**		
Have you ever heard or read anything about Dementia or Alzheimer's? [awareness of dementia]	Checkboxes	Yes l No
Do you know what Dementia or Alzheimer's is? [knowledge on dementia]	Checkboxes	Yes l No
- If yes [question before]: What is Dementia or Alzheimer's? (You can tick more than one)	Checkboxes, free text field	Process of normal ageing l Disease of older age l Can also affect younger people l Witchcraft l Others [free field]
Socio-demographic characteristics of grandparents:		
- Age of your grandparents*	Free text field	Age in years
- Are your grandparents* dead or alive?	Checkboxes	Dead l Alive
- Did your grandparents* have trouble with memory loss?	Checkboxes	Yes l No
Did a person in your family suffer from symptoms like memory loss or were they unable to perform tasks of daily living?	Checkboxes, free text field	Yes l No<br>If yes: age in years

**Data on the belief in witchcraft**		
Do you believe in witchcraft?	Checkboxes	Yes l No
Do you think witchcraft works?	Checkboxes	Yes l No
Do you know people in your family who believe in witchcraft?	Checkboxes	Yes l No
Did you ever meet people that were accused of witchcraft?	Checkboxes	Yes l No
Do you think that witchcraft is an important part of your culture?	Checkboxes	Yes l No
Which of the following situations and behaviors would you associate with witchcraft? (You can tick more than one!)	Checkboxes	Roaming around on the street l Memory loss l Forgetfulness l Confused speech l Swearing at others
**Data on future perspectives concerning older people, age-related diseases, and witchcraft**		
Do you think older people (aged 60+) need more attention in Ghana?	Checkboxes, open question	Yes l No<br>Please explain [free text field]
Do you think age-related diseases need more attention in Ghana?	Checkboxes, open question	Yes / No<br>Please explain [free text field]
Do you think witchcraft needs more attention in Ghana?	Checkboxes, open question	Yes / No<br>Please explain [free text field]

* Answers could be given for each grandparent: grandmother (paternal side); grandfather (paternal side); grandmother (maternal side); grandfather (maternal side)
