# Supplementary material for: Dementia Awareness Challenges in Sub-Saharan Africa: A Cross-Sectional Survey Conducted Among School Students in Ghana
Source: Am J Alzheimers Dis Other Demen. 2022 Jan 5;36:15333175211055315. doi: 10.1177/15333175211055315 (PMC10581119; doi:10.1177/15333175211055315)
Supplement: sj-pdf-2-aja-10.1177_15333175211055315 – Supplemental Material for Dementia Awareness Challenges in Sub-Saharan Africa: A Cross-Sectional Survey Conducted Among School Students in Ghana [file sj-pdf-2-aja-10.1177_15333175211055315.pdf]

Supplementary File 2. Respondents' statements related to the lack of awareness of aging or age-related disease.

Why do you think older people need more attention in Ghana?	Why do you think age-related diseases need more attention in Ghana?
The people of Ghana need to be informed about the aging process and its challenges.	Because there is a lack of general awareness of age-related diseases in Ghana.
Because some people in Ghana are not educated on the process of ageing.	So that people in Ghana can become aware of age-related diseases.
Because older people are physically, mentally, and spiritually weak.	More attention will make people in Ghana aware of the diseases related to age.
Old people are mostly considered witches.	Because many people in Ghana have no knowledge on age-related diseases.
Most older people are accused of being a witch/wizard, which they are not.	Because people in Ghana need to be educated on age-related diseases.
Because older people are harmless.	Increased attention to age-related diseases would help to better understand the diseases and improve knowledge on the diseases.
Because due to illiteracy some people in Ghana think old people are witches/wizards.	More attention is needed to create awareness of various diseases of old age.
Because people in Ghana think that people aged 60 years and older are witches/wizards.	Because knowledge on age-related diseases would help to treat or cure people living with such diseases.
Because people who are 60 years and older are believed to use witchcraft.	More attention would help to understand the behavior of people with age-related diseases.
Due to menopause, older women act in a different way so people in Ghana think they are witches.	Many people in Ghana do not know the impact that age-related diseases have on life.
Because old people are seen as being affected by witchcraft.	More attention is needed because people with age-related diseases are sometimes victimized by others in society who believe in witchcraft.
Elderly people no longer have a job or work and live in the world of witchcraft.	People should be educated that witchcraft is not responsible for the illness.
Old people are accused of witchcraft, so they need more attention.	People in Ghana living with age-related illnesses should continue to be perceived as persons.
Because older people are usually branded as witches and wizards, and they mostly take care of themselves without help.	Because most elderly people are regarded as witches.
Because most of the elders are humiliated and accused of witchcraft.	Age-related diseases may be considered a spiritual matter rather than a health issue.
Because older people are mostly accused of witchcraft.	Ghanaians classify people living with age-related illnesses as witches and wizards.
Because older people will always be the victims of witchcraft.	Because people in Ghana say age-related diseases are caused by witchcraft.
Because usually the older ones become weak at some point and can then be easily abused.	Because people in Ghana tend to think if you die young, maybe you were bewitched.
The behavior and actions of old people are like those of children, therefore older people need more attention.	To decrease the issues of witchcraft in Ghana.
Because when people get older their ability of thinking is reduced.	So that society cannot attribute the behavior of people with age-related diseases to the madness of witchcraft.
Because the mind of older people is like that of children.	So that learning can eliminate the myths in the minds of people in Ghana.
Older people are very dangerous and normally behave like children.	Many people think old people are witches but mostly all they need is love.

A free field allowed respondents to justify their answers to the questions, *"Do you think older people need more attention in Ghana?"* and *"Do you think age-related diseases need more attention in Ghana?"* Statements regarding a lack of awareness of the process of aging and age-related diseases were included in this table.
